# Immunoblot screening of CRISPR/Cas9-mediated gene knockouts without selection

**DOI:** 10.1186/s12867-016-0061-0

**Published:** 2016-04-02

**Authors:** Jason A. Estep, Erin L. Sternburg, Gissell A. Sanchez, Fedor V. Karginov

**Affiliations:** Department of Cell Biology and Neuroscience, Institute for Integrative Genome Biology, University of California, Riverside, CA 92521 USA

**Keywords:** CRISPR/Cas9, Clonal selection, Screening, Dot blot

## Abstract

**Background:**

Targeted genomic editing using the CRISPR/Cas9 methodology has opened exciting new avenues in probing gene function in virtually any model system, including cultured mammalian cells. Depending on the desired mutation, several experimental options exist in the isolation of clonal lines, such as selection with introduced markers, or screening by PCR amplification of genomic DNA. However, streamlined approaches to establishing deletion and tagging mutants with minimal genomic perturbation are of interest in applying this methodology.

**Results:**

We developed a procedure for rapid screening of clonal cell lines for the deletion of a protein of interest following CRISPR/Cas9 targeting in the absence of selective pressure based on dot immunoblots. To assess the technique, we probed clonal isolates of 293-TREx cells that were targeted with three separate sgRNAs against the HuR gene. Validation of knockout candidates by western blot indicated that the normalized protein abundances indicated by the dot blot serve as accurate predictors of deletion. In total, 32 independent biallelic deletion lines out of 248 screened clones were isolated, and recovery of null mutants ranged from 6 to 36 % for the individual sgRNAs. Genomic sequencing verified small deletions at the targeted locus.

**Conclusions:**

Clonal screening for CRISPR/Cas9-mediated editing events using dot immunoblot is a straightforward and efficient approach that facilitates rapid generation of genomic mutants to study gene function.

## Background

Manipulating protein levels and activities is a principal tool in understanding the functions and the relationships of these molecular components in cells. Transient perturbations involve chemical inhibition (and activation) with small molecules, overexpression from non-integrating vectors, or knockdown by RNA interference [1]. Often more experimentally desirable, stable genetic alteration can be achieved by integration of overexpression and shRNA constructs [2], or by genomic editing of the endogenous protein locus with transcription activator-like effector nucleases (TALENs) [3], zinc-finger nucleases [4, 5], and, more recently, by RNA-guided nucleases based on the clustered, regularly interspaced short palindromic repeats (CRISPR)/Cas9 system [6–11]. Combinations of stable integration and modification with temporal control include the use of chemically inducible systems, such as tetracycline repression/activation [12, 13], tamoxifen control of Cre-ER recombination [14, 15], and more modular control of expression, localization and activity by small molecules [16–18].

**Fig. 1:**
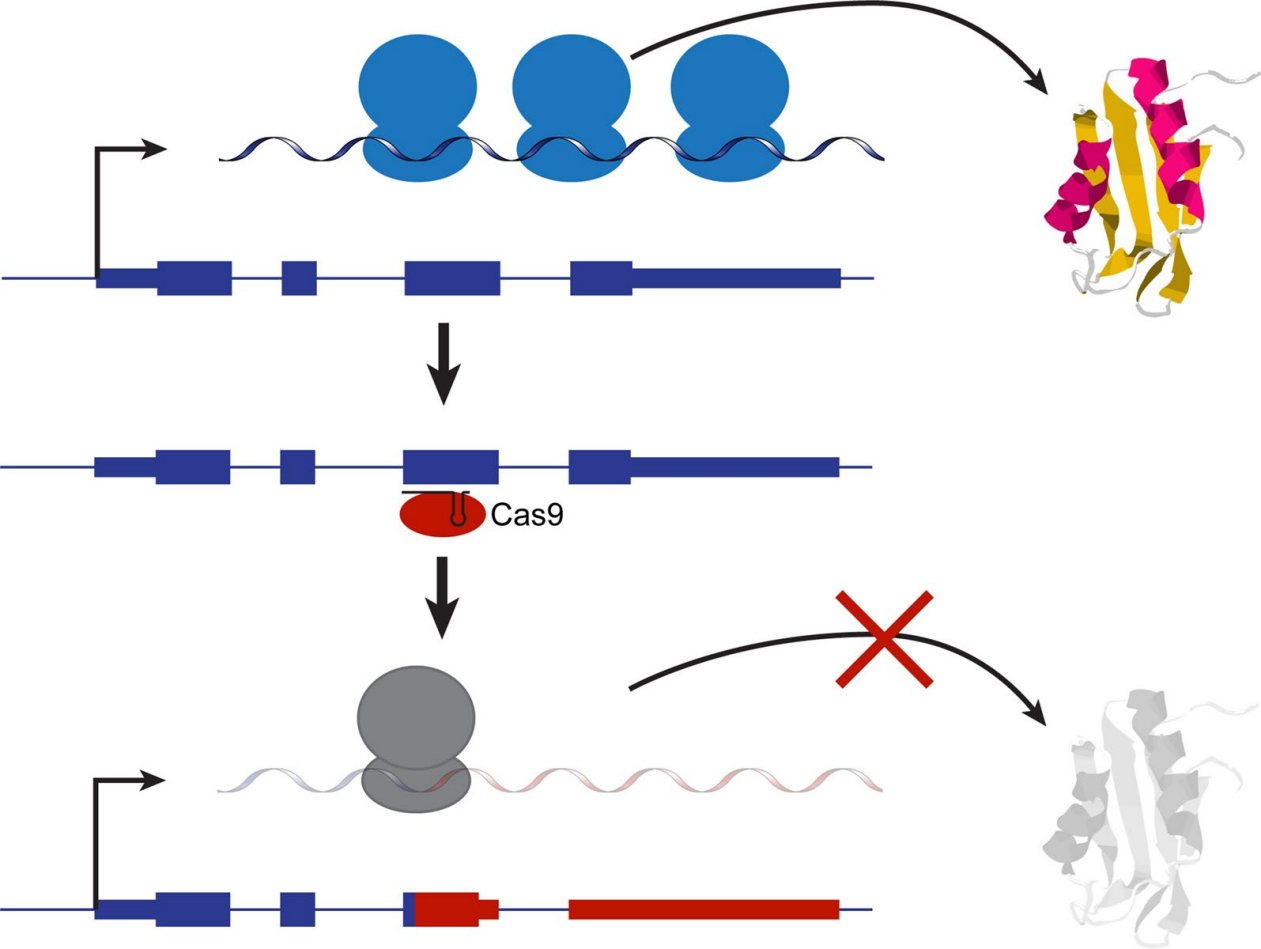
sgRNA/Cas9-mediated gene knockouts. sgRNA/Cas9 targeting to a gene exon induces a double-stranded DNA break that is repaired by non-homologous end joining, often introducing frameshift mutations (depicted in *red*) that lead to lack of functional protein production and likely cause nonsense-mediated decay of the mRNA

The CRISPR/Cas module is an endogenous adaptive immunity system commonly used by bacteria and archaea to counteract phage infection and introduction of plasmid DNA [19–22]. Short (20–30 bp) sequence tags from the invaders are incorporated as spacers between direct repeats of a CRISPR locus. Its transcription and processing yields small crRNAs that associate with and guide CRISPR-associated (cas) protein(s) to complementary DNA targets for endonucleolytic cleavage. Due to its particularly simple makeup, the type II CRISPR/Cas system of *Streptococcus pyogenes* has been adapted for genomic editing with great success: a single protein, Cas9, is required for crRNA binding and cleavage, and the RNA components have been engineered into a single guide RNA (sgRNA) [23]. Thus, targeting of nearly any genomic sequence is possible with the introduction of an sgRNA and Cas9 into the cells of interest [24, 25]. In its simplest form, creation of a knockout line depends on the sgRNA-guided dsDNA cleavage by the Cas endonuclease, followed by non-homologous end joining (NHEJ) repair of the site by the cell. A small, random deletion is often introduced at the repair site. Deletions that target the open reading frame and result in frame-shifts, particularly early in the mRNA, are very likely to yield a non-functional protein sequence and to target the mRNA for nonsense-mediated decay due to premature stop codons in the new frame (Fig. 1). In contrast, small in-frame deletions may not compromise the function of the protein. Aside from NHEJ-mediated deletions, the Cas cleavage can also stimulate homology-driven repair based on a supplied DNA template, and allow for larger deletions or insertion of tags and markers.

In the targeted modification strategies, the researcher has the option to isolate correct clones by introducing a selectable marker at the genomic locus. While this approach is beneficial if the sgRNA has low efficiency, or if the mutation confers a strong selective disadvantage to the cells, it requires the construction of repair template vectors containing the marker and homology arms specific to the locus [26]. Furthermore, it leaves a significantly-sized, transcribed insertion that may affect the expression of nearby genes, although it may be excised through an additional Cre-mediated step, if surrounded by loxP sites [27].

However, the relatively high efficiency of Cas9 targeting allows for clonal screening without selection. In a common approach, purification of genomic DNA from isolated clones permits PCR amplification followed by sequencing, deletion analysis or restriction digest [28]. In the present study, we describe a simple method for isolation of clonal deletion mutants based on CRISPR/Cas9 targeting combined with dot immunoblot analysis and validated by western blots and DNA sequencing. Screening directly for protein production ensures proper knockout, avoiding signal from in-frame deletions/mutations that may not eliminate its function. The approach can also be applied to screening for insertions of tags mediated by homologous recombination.

## Results and discussion

We wished to apply the CRISPR/Cas system to establish stable cell lines completely lacking specific protein function. As with other genetic mutant approaches, it is beneficial to isolate several independently derived knockout clones to control for secondary mutations. In the case of CRISPR/Cas, off-target cleavage events at sites of imperfect sgRNA complementarity are known to occur [29, 30], and the use of different sgRNAs can mitigate the possibility of spuriously observed phenotypes. We used pre-designed human genomic target sites for the *S. pyogenes* Cas9 effector nuclease, picking the reduced set of three sites closer to the 5′ end of the gene [31]. The targeted sites were introduced into sgRNA-producing context in a plasmid that also co-expresses the Cas9 protein, pSpCas9(BB) [24, 28].

**Fig. 2:**
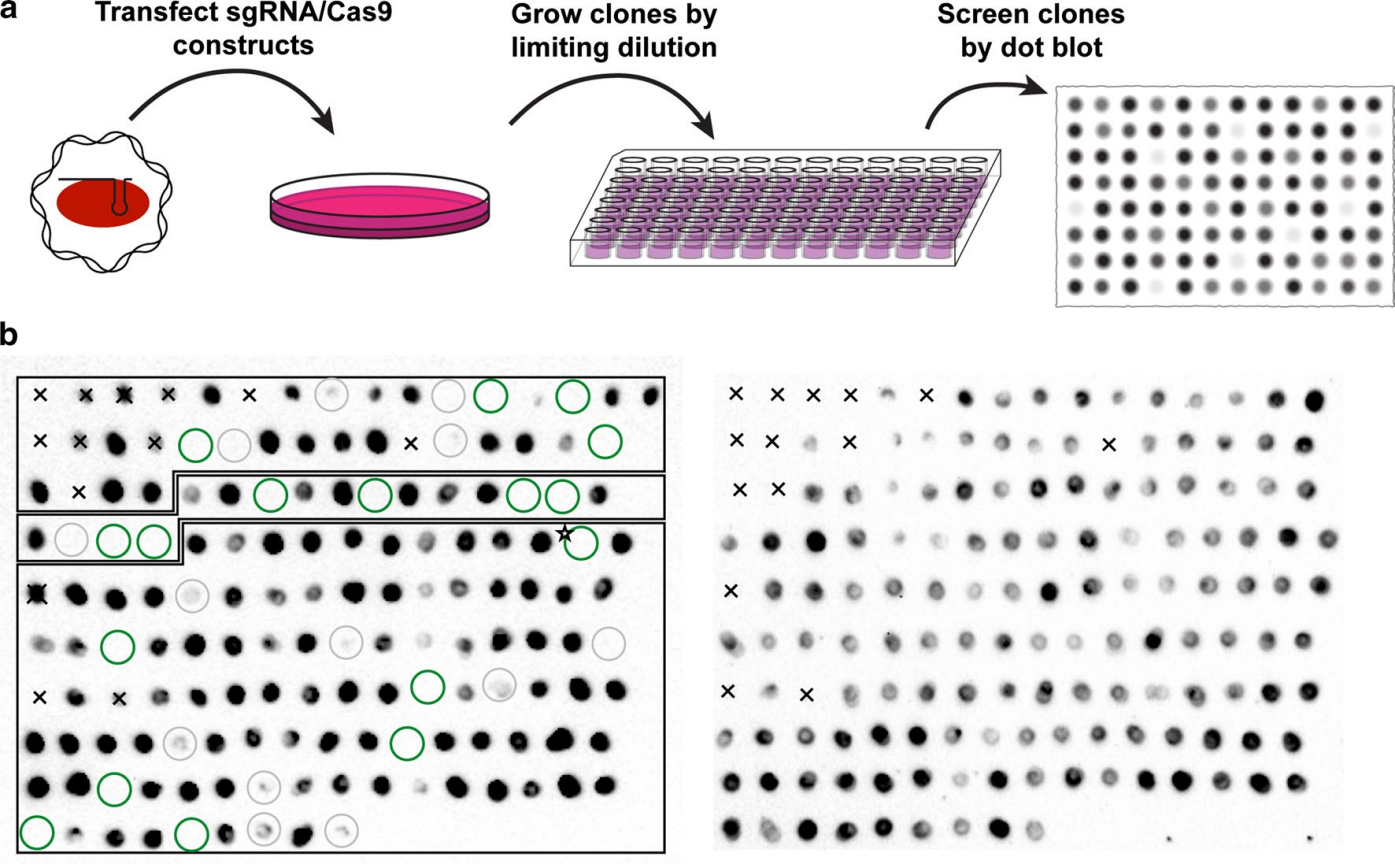
Identification of knockout lines by protein dot blot. **a** Schematic of the procedure: after transfection, cells are plated at limiting dilution in 96-well plates, and isolated colonies are picked and expanded. Lysates from clonal populations are blotted on a nitrocellulose membrane and probed for the protein of interest, along with a parallel control protein blot. **b** One batch of isolated clones (from a total of two) is shown after dot blotting with HuR antibody (*left*), and separate blotting for a control protein, Pum2 (*right*). Clones with insufficient control protein signal (<two fold of the membrane background), denoted by an x, likely arose due to pipetting or blotting error, and were excluded from further analysis. *Circles* denote clones that validated (*green*) or did not validate (*grey*) by subsequent western blot. Clone HuR-5.B5 is designated by a star. Boxes around the blots indicate clones isolated from HuR-3, 4, and 5 targeting constructs (from *top* to *bottom* respectively)

We opted to develop a simple, selection-free strategy based on dot blots for the protein of interest to isolate the knockout clones (Fig. 2a). First, a bulk population of cells is transfected with the sgRNA/Cas9 constructs. To ensure a high percentage of expressing cells, the transfection procedure may be sequentially repeated, and a parallel control transfection with a GFP marker is performed. Next, transfected cells are plated at limiting dilution concentrations into 96-well plates, and clonal populations are established. Setting aside a propagating aliquot for each, confluent clones are lysed in the plate with a passive lysis buffer. To identify knockout candidates, a small amount of lysate (1 µl) is blotted onto two nitrocellulose membranes, to be immunoblotted for the protein of interest, and a normalization control. Alternatively, a single membrane may be probed sequentially. Clones that exhibit little or no expression of the protein, while demonstrating a measurable amount of cell extract in the control blot, can be identified visually, or by quantifying the ratio of the corresponding dot intensities. In this fashion, screening directly for protein production ensures a functional knockout, as opposed to deletions/mutations that may not efficiently disrupt the protein. It should be noted that the extent of cross-reactivity of the antibody will affect the accuracy of the method, since genuine knockout lines may still display a significant level of background signal from other proteins in the blot.

We applied the above strategy to generate a knockout of the HuR (ELAVL1) protein in 293-TREx cells, targeting exons 3, 4, and 5 with three distinct sgRNAs. Most of the expanded clones exhibited parental growth rates and morphology, while several lines grew substantially slower and/or with altered cell shape. However, these features did not correlate with the absence of HuR (see below), likely reflecting spurious mutations acquired due to CRISPR/Cas off-targeting or simply during the procedure. Thus, HuR is dispensable to normal growth rates under optimal conditions in 293-TREx cells; however, if the protein of interest is expected to affect the division or morphology of cells, it can form the basis of a phenotypic pre-screen during clonal expansion. Mutations that are substantially detrimental to growth rates can still be recovered, since the clonal isolation begins soon after the introduction of the sgRNA/Cas9.

**Fig. 3:**
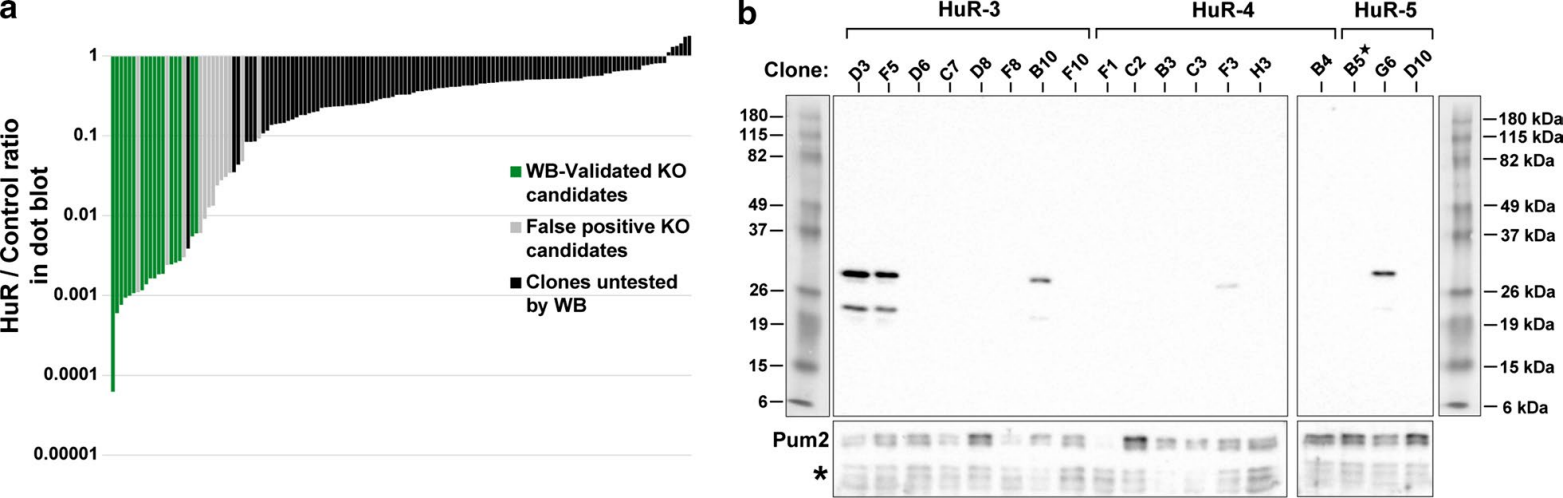
Quantification and validation of KO candidates. **a** Ratios of HuR to control protein signal in the dot blot shown in Fig. 2b, in increasing order. Clones that were confirmed or were not confirmed by western blot are shown in *green* and *grey*, respectively. **b** Western blot for HuR (*top panels*) of a subset of clones identified by dot blot. *Bottom panel* shows a control blot for Pum2 of the same membrane. Clone HuR-5.B5 is designated by a *star*

Isolated clones were grown and tested for the presence of HuR with a dot blot (Fig. 2b, left), yielding several candidates with complete or near-complete lack of HuR (circled). In addition to visual inspection, HuR expression signal on the dot blots was quantified relative to a loading control (Fig. 2b, right). After eliminating clones with low control expression (less than twofold of the average background staining of the blot), the ratios displayed a range of >10^4^-fold in normalized HuR expression (Fig. 3a). A possible expectation was for the HuR levels to cluster into stepwise values representing wild-type, heterozygous, and homozygous mutant clones. Instead, the observed levels form a fairly smooth gradient, likely due to clonal variability in expression, as well as probable compensatory mechanisms affecting expression in heterozygotes. Of note, our estimates based on absolute protein quantification in the literature [32] indicate that ~300 fmol (11 pg) of HuR were blotted, and we expect that substantially lower protein amounts should be reliably detected, provided suitable antibodies are available.

**Fig. 4:**
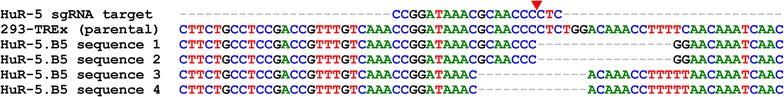
Sequence of the genomic deletions in clone HuR-5.B5. The genomic region surrounding the targeted site was PCR-amplified from wt 293-TREx cells and clone HuR-5.B5, and placed into a plasmid vector. Individual plasmid clones were sequenced. The Cas9 cleavage site is denoted by a *red triangle*

Forty four of the 248 identified candidates (examples shown in Fig. 2b, circles), including some with low but visible signal in the dot blot, were further tested for the absence of HuR by western blot (example in Fig. 3b). In an all-or-none fashion, 32 clones validated as complete knockouts, while 12 appeared to be false positives. The false positives may have resulted from pipetting error during blotting; from stochastically low, but non-zero HuR expression in some wild-type clones; and from selecting clones with comparatively fewer cells in the lysate (observed as low expression both for HuR and the control). Indeed, the validated KO lines had significantly lower normalized HuR signal (this remaining signal likely arising from antibody cross-reactivity) relative to the candidates that were not confirmed (Fig. 3a). This indicated that the HuR/control ratio is an accurate quantitative criterion in separating true knockouts from wild-type clones in our broad set of tested candidates, and can serve as a reliable selection filter. It should be noted that a modification to the procedure that would strip and re-probe the same blot for both proteins could eliminate some of the signal variability, at the expense of a slightly lengthier protocol. Finally, we verified the deletions in clone HuR-5.B5 by amplifying, cloning and sequencing the corresponding genomic region (Fig. 4). Four sequenced clones revealed two different short deletions, consistent with independent NHEJ events at two chromosomal locations. In both cases, the deletions occurred near the Cas9 cleavage site three nucleotides upstream of the protospacer-adjacent motif (NGG) [23, 33], as observed previously [24].

The effectiveness of generating homozygous knockouts varied substantially with the sgRNA used (Table 1), ranging from 6 to 36 % of the initially tested clones. Knockout production was reproducible, as an independent replicate of the experiment with guide RNAs 3 and 4 yielded 10 and 8 % of null mutations respectively (data not shown), with the variability likely arising from transfection efficiencies. Considering that these lines represent concurrent editing of both alleles, and most likely require a frameshift mutation, the observed KO rates compare favorably with the ~6–50 % rates of overall NHEJ-driven indels measured by sequencing or the SURVEYOR assay using similar delivery methods [34, 35]. The variability among the three tested sgRNAs also agrees with related observations for multiple guide RNAs at a given locus [24], presumably reflecting differences in sgRNA production, complex formation with Cas9, and targeting/cleavage efficiency [36]. However, CRISPR/Cas editing efficiencies may also show loci-specific variability, potentially explained by the dependence of Cas9 binding on chromatin accessibility, reflected in DNase hypersensitivity and CpG methylation [37, 38]. Nevertheless, screening of a reasonably small number of initial clones is likely to produce more than one independent deletion mutant using this simple procedure.

**Table 1:** Number of analyzed and validated KO clonal lines for the sgRNAs used

SgRNA construct	# of clones tested by dot blot	# of validated KOs	KO percentage (%)
HuR-3	69	15	22
HuR-4	22	8	36
HuR-5	157	9	6

Aside from targeting individual genes for knockout, the CRISPR/Cas system allows many additional means of genome editing. We assessed whether our screening protocol can be used to achieve simultaneous knockout of tandemly arranged genes by inducing large chromosomal deletions between two sgRNA target sites. Small deletions have been previously achieved without selection [24], while larger biallelic and monoallelic deletions have been created after sorting for strong Cas9 expression [39], or in haploid embryonic stem cells [40]. In our hands, attempts to excise the ~250 Kbp human Ago4, 1, 3 locus by targeting Ago4 and Ago3 while blotting for Ago1 have not yielded any complete deletions (data not shown). Thus, elimination of large genomic fragments may require the use of homologous recombination templates spanning the deletion, enrichment for cells expressing Cas9 (without genomic insertion), and/or incorporation of selectable markers at the deletion site. Similarly, gene mutagenesis without deletion would require homologous templates involving selection or protein tagging. In these cases, dot blotting for the tag may still be used as a screening step. It should be pointed out that directly blotting for the protein under study is limited by antibody availability, and assaying of secreted proteins may necessitate the collection of culture media instead of cell pellets.

## Conclusions

The CRISPR/Cas system is transforming modern genetics and molecular biology by offering unprecedented ways of genomic manipulation. We have described a methodology to isolate simple deletion mutants of a gene of interest by CRISPR/Cas based on dot-blot screening for the resulting protein product, without the need for intervening selection steps. The advantages of the method lie in the ease of upfront preparation (only the sgRNA constructs need to be created, based on short synthetic oligonucleotides), the relative simplicity of the screening procedure, and the minimal genomic perturbations to the resulting clonal lines. For a typical knockout experiment, our estimates indicate a time saving of 4–5 days relative to direct western blotting, or on the order of weeks relative to design and construction of selection steps. The protocol can be modified to allow for editing with homologous recombination templates, by blot screening for protein tags introduced on the template. The presented techniques should expand the available toolkit for the application of biochemistry and molecular and cell biology approaches to the study of protein function in mammalian cell culture.

## Methods

### Preparation of Cas9-sgRNA plasmids

The pSpCas9(BB) plasmid was a gift from Feng Zhang (Addgene plasmid #42230). Single guide RNA sequences were constructed to target the pre-designed loci in the third, forth, and fifth exon of the HuR gene (HuR-3, TGTGAACTACGTGACCGCGA;HuR-4,CGGGCGAGCATACGACACCT;HuR-5,CCGGATAAACGCAACCCCTC) and cloned into the parental plasmid as previously described [28]. Clones were tested for correct inserts using Sanger sequencing.

### Cell Culture, transfections, and dilutions

293-TREx cells were grown in Dulbecco’s Modification of Eagle’s Medium (DMEM) with 4.5 g/L glucose, l-glutamine and sodium pyruvate (Corning Cellgro Ref:10-013-CV) that was supplemented with 10 % Fetal Bovine Serum (HyClone FBS Characterized Cat: SH30071.03) and 10 units/ml of Penicillin–Streptomycin (HyClone Cat: SV30010). Cultured plates were grown in a humidified incubator supplying 5 % CO2 at 37 °C.

Two independent transfection events were performed for each construct. First, cells were grown in a 6-well format to approximately 60 % confluency and transfected with 2.5 µg of the prepared Cas9-sgRNA plasmid using the calcium phosphate method. 24 h later cells were passaged into 10 cm plates. 24 h following passaging, cells were again transfected with 12.5 µg plasmid using the TransIT-LT1 reagent (Mirus, Cat: MIR 2300). Cells were allowed to reach 100 % confluency before seeding out into individual clones. After the transfections, efficiency was estimated to be 70–80 % by GFP expression in a parallel transfection with a modified version of the pMSCV-PIG vector.

To isolate individual clones, cells were seeded at a density of 3–5 cells per 96-well. Colony growth in less that 60 % of the wells was observed over 3–4 weeks, and media was changed weekly to prevent drying of wells. Clone colonies that were visible to the eye were picked into 96-well plates by aspirating the media, dispensing 2 µl of fresh media onto the colony, scraping across the colony with the pipette tip, and drawing the media back. 24–48 h later, isolated clones that were growing as a clump were reseeded by pipette mixing in the well to encourage monolayer cell growth.

### Dot blot and western blot

Individual clones were grown until 100 % confluency and collected for dot blot analysis, dislodging the monolayer of cells by pipeting within the well. 90 µl of the 100 µl total volume was then removed, spun down, and the cell pellet lysed in 10 µl of 1× Passive Lysis Buffer (Promega’s Dual-Luciferase Reporter Assay System, Cat: E1910). The remainder of the cells were propagated. 1 µl of cell lysate was pipetted onto dry nitrocellulose membrane (Bio-Rad, Cat: 162-0115) to form a dot. Each sample was blotted twice on two separate membranes, creating two identical patterns of samples, blocked in 5 % milk in TBST for 1 h at room temperature, and blotted for HuR (Santa Cruz Biotechnology, clone 3A2, Cat: sc-5261, 1:1000–1:5000 dilution) and Pum2 (Bethyl Labs, Cat: A300-202A, 1:1000 dilution) in 5 % milk in TBST for 1 h, with 3 × 5 min TBST washes in between incubation steps. Anti-Mouse-HRP-conjugated secondary antibody was from Cell Signaling (anti-mouse, Cat: 7076). Anti-Rabbit-HRP-Conjugated Secondary Antibody was from Cell Signaling (anti-rabbit, Cat: 7074). Blotted membranes were imaged on a Biorad ChemiDoc MP imager and quantified by Image Lab: identical circles were centered on each dot, and the signal volume was computed by the software. Local background values for each dot were computed and subtracted by Image Lab. Western blotting was performed as described above.

### Sequencing of genomic DNA

To amplify a genomic fragment flanking the HuR-5 target region, primers were designed containing BbsI restriction sites with distinct overhangs (HuR-5 For, CGACTTGAAGACCTCACCTGTGATGGGCTCAGAGGAACC; HuR-5 Rev, CGACTTGAAGACCTAAACAGACTTCTAGCCTGGCCCAC). Genomic DNA of the parental 293-TREx line and the HuR-5.B5 mutant was isolated by standard SDS/Proteinase K lysis followed by phenol/chloroform extraction and isopropanol precipitation. The target region was PCR amplified using Q5 High Fidelity DNA Polymerase (New England Biolabs, Cat:M0491S), and cloned by Golden Gate cloning into a modified pRL-TK plasmid bearing two consecutive BbsI sites (ThermoFisher Scientific Cat# FERFD1014). Isolated bacterial colonies were mini-prepped and Sanger sequenced.

## Abbreviations

CRISPR: clustered, regularly interspaced short palindromic repeats
sgRNA: single guide RNA
shRNA: short hairpin RNA
TALEN: transcription activator-like effector nuclease
crRNA: CRISPR RNA
NHEJ: non-homologous end joining
